# MyD88 regulates a prolonged adaptation response to environmental dust exposure-induced lung disease

**DOI:** 10.1186/s12931-020-01362-8

**Published:** 2020-04-22

**Authors:** Amber N. Johnson, Jack R. Harkema, Amy J. Nelson, John D. Dickinson, Julianna Kalil, Michael J. Duryee, Geoffrey M. Thiele, Balawant Kumar, Amar B. Singh, Rohit Gaurav, Sarah C. Glover, Ying Tang, Debra J. Romberger, Tammy Kielian, Jill A. Poole

**Affiliations:** 1 Department of Internal Medicine, University of Nebraska Medical Center, 985990 Nebraska Medical Center, Omaha, NE 68198-5990 USA; 2 Pathobiology & Diagnostic Investigation, Institute for Integrative Toxicology, College of Veterinary Medicine, Michigan State University, East Lansing, MI USA; 3 Veterans Affairs Nebraska-Western Iowa Health Care System, Research Service, Omaha, NE USA; 4 Department of Biochemistry and Molecular Biology, University of Nebraska Medical Center, Omaha, NE USA; 5 Department of Medicine, University of Mississippi Medical Center, Jackson, MS USA; 6 Department of Medicine, University of Florida, Gainesville, FL USA; 7 Department of Microbiology and Pathology, University of Nebraska Medical Center, Omaha, NE USA

**Keywords:** Environmental respiratory and skin disease, Agriculture, Occupational, Organic dust, Airway inflammation, Adaptation, MyD88

## Abstract

**Background:**

Environmental organic dust exposures enriched in Toll-like receptor (TLR) agonists can reduce allergic asthma development but are associated with occupational asthma and chronic bronchitis. The TLR adaptor protein myeloid differentiation factor88 (MyD88) is fundamental in regulating acute inflammatory responses to organic dust extract (ODE), yet its role in repetitive exposures is unknown and could inform future strategies.

**Methods:**

Wild-type (WT) and MyD88 knockout (KO) mice were exposed intranasally to ODE or saline daily for 3 weeks (repetitive exposure). Repetitively exposed animals were also subsequently rested with no treatments for 4 weeks followed by single rechallenge with saline/ODE.

**Results:**

Repetitive ODE exposure induced neutrophil influx and release of pro-inflammatory cytokines and chemokines were profoundly reduced in MyD88 KO mice. In comparison, ODE-induced cellular aggregates, B cells, mast cell infiltrates and serum IgE levels remained elevated in KO mice and mucous cell metaplasia was increased. Expression of ODE-induced tight junction protein(s) was also MyD88-dependent. Following recovery and then rechallenge with ODE, inflammatory mediators, but not neutrophil influx, was reduced in WT mice pretreated with ODE coincident with increased expression of IL-33 and IL-10, suggesting an adaptation response. Repetitively exposed MyD88 KO mice lacked inflammatory responsiveness upon ODE rechallenge.

**Conclusions:**

MyD88 is essential in mediating the classic airway inflammatory response to repetitive ODE, but targeting MyD88 does not reduce mucous cell metaplasia, lymphocyte influx, or IgE responsiveness. TLR-enriched dust exposures induce a prolonged adaptation response that is largely MyD88-independent. These findings demonstrate the complex role of MyD88-dependent signaling during acute vs. chronic organic dust exposures.

## Background

Environmental agriculture organic dust exposures are rich in Toll-like receptor (TLR) agonists that are associated with regulating airway allergic and non-allergic inflammatory diseases [1]. Early life exposures to these TLR-enriched environments appears to be protective against the development of IgE-mediated diseases, including eosinophilic asthma [2–4]. However, workplace exposure to these organic dusts is associated with increased occupational asthma, workplace exacerbated asthma, neutrophil-predominant pulmonary inflammation, chronic bronchitis, and chronic obstructive pulmonary disease (COPD) [2, 5–7]. This dichotomy implies that persistent exposures to these environmental inflammatory-inducing agent(s) modulate the lung inflammatory response with potential long-term consequences.

The complexity and biodiversity of agriculture dust exposures is increasingly recognized, and animal and human studies have defined roles for TLR2/TLR4/TLR9-signaling pathways [8–12]. The TLR/IL-1R/IL-18R adaptor protein myeloid differentiation factor 88 (MyD88) that is used by all TLRs except for TLR3 [13] has been shown to have a fundamental role in the acute inflammatory response to organic dust [10, 14, 15] as well as other inflammatory exposures [16, 17]. MyD88 signaling mediates bleomycin-induced IL-17B expression in alveolar macrophages to promote pulmonary fibrosis [16]. In silica-induced fibrosis, silica dust increases MyD88 expression in macrophages [17]. In experimental asthma, MyD88 expression in epithelial cells mediates eosinophilia whereas MyD88 expression in conventional dendritic cells controls the neutrophilic response [18]. In response to agriculture organic dust extract (ODE), MyD88 knockout (KO) mice demonstrate reduced airway hyper-responsiveness and near absence of neutrophil influx and inflammatory cytokine release following acute ODE exposure [10]. However, these animals demonstrate an elevated, as opposed to reduced, mucus metaplasia response to acute ODE challenges [14]. Despite the well-characterized role for MyD88 in mediating acute responses to ODE, its impact on airway inflammatory responses to repetitive, prolonged exposures is unknown. This information could be important in future targeted approaches.

Repetitive exposures with ODE from large animal farming facilities induces perivascular lymphocytic aggregates that slowly, but not completely, resolve in size and number by 4 weeks after the inflammatory insult is removed [19]. It is not known if there is a heightened or dampened airway inflammatory response upon re-exposure. In other settings, endotoxin exposure can induce a refractory state of hypo-responsiveness for several days/weeks [20]. Alternatively, the lung microenvironment could be poised to be hyper-responsive to rechallenge, which collectively would have implications for workers returning to the job site after prolonged absences. It is unclear whether MyD88 signaling is involved in rechallenge responses.

In this study, we sought to investigate the lung cellular and microenvironment response to repeated ODE exposure for 3 weeks and to determine whether prolonged recovery (i.e. 4 weeks) following repetitive exposures would result in a heightened or refractory response to subsequent ODE rechallenge. Moreover, we hypothesized that the MyD88 signaling pathway would be central to governing lung responses to ODE. Collectively, our findings demonstrate a prominent role for MyD88 in mediating repetitive ODE-induced airway inflammatory responses, including an adaptive role for regulating the expression of epithelial barrier integral proteins. Moreover, our results also suggest that repetitive ODE treatment heightens neutrophil influx but decreases inflammatory cytokine release, while simultaneously upregulating an anti-inflammatory and macrophage response following a later ODE insult. These studies highlight a contextual role of MyD88-dependent signaling in orchestrating the pulmonary inflammatory response to acute and repetitive organic dust exposures.

## Methods

### Animals

MyD88 gene knock-out (KO) mice on a C57BL/6 J background were provided by S. Akira (Okasa, Japan). C57BL/6 J wild-type (WT) mice from The Jackson Laboratory (Bar Harbor, ME) were used as controls. Mice were fed alfalfa-free chow ad libitum (Harlan Laboratories) as recommended by The Jackson Laboratory. Mice were group housed in an SPF (specific-pathogen-free) facility under 12- h light and dark cycles. All animal procedures were approved by the Institutional Animal Care and Use Committee at the University of Nebraska Medical Center and conducted according to the U.S. National Institutes of Health guidelines for the use of rodents. Male and female mice aged 6–12 weeks were utilized in experimental procedures.

### Organic dust extract

Aqueous organic dust extract (ODE) was prepared from settled dust collected from horizontal surfaces 3 ft. above the floor in swine confinement feeding operations. Extracts were batch prepared and filter (0.22 μm) sterilized as previously described [21, 22]. Stock ODE was diluted to a concentration of 12.5% (vol/vol) in sterile phosphate buffered saline (PBS, pH 7.4, diluent), a concentration previously shown to elicit optimal lung inflammation in mice [21, 22]. ODE 12.5% contains approximately 3–4 mg/ml total protein as measured by Nanodrop spectrophotometry (NanoDrop Technologies, Wilmington, DE) and endotoxin levels range from 22.1 to 91.1 EU/ml as measured by the limulus amebocyte lysate assay using manufacturer instructions (Sigma). Specific microbial biomarkers inferred from shotgun metagenomics of DNA pyrosequencing reads of the dust samples have been previously detailed [23].

### Murine ODE exposure model

**Fig. 1:**
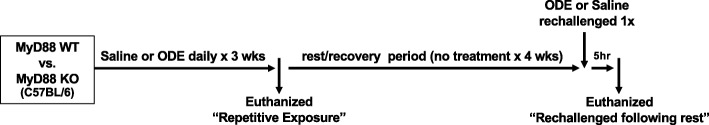
Experimental design protocol. MyD88 WT and knockout (KO) mice were intranasally treated with saline or ODE daily for 3 weeks and then were euthanized (“repetitive exposure”) or allowed to rest for 4 weeks withouat any treatments (“rest/recovery period”) and subsequently rechallenged once with either saline or ODE and euthanized (“rechallenged following rest”)

Under light sedation with isoflurane inhalation, mice were administered 50 μl of 12.5% ODE or sterile saline (PBS) by intranasal inhalation every day for 3 weeks with weekends excluded as previously described [21, 22]. Mice were euthanized 5 h after final exposure for experimental endpoints (Fig. 1). In separate studies, mice were treated with saline or ODE daily for 3 weeks (“repetitive exposure”) and then allowed to rest for 4 weeks without any treatments (“rest/recovery phase”) and subsequently rechallenged once with either saline or ODE and euthanized 5 h following this final rechallenge exposure for experimental endpoints (Fig. 1).

### Bronchoalveolar lavage fluid studies

Bronchoalveolar lavage fluid (BALF) was collected by instilling 3 × 1 ml PBS into the airway. Cytokines implicated in agriculture respiratory disease including IL-6, neutrophil chemoattractants CXCL1 and CXCL2, and TNF-α [1] were measured in the cell-free supernatant of the first lavage by ELISA (R&D Systems, Minneapolis, MN) with limits of detectability of 1.8, 2.0, 1.5, and 7.2 pg/ml, respectively. Total number of cells recovered from pooled lavages were enumerated and differential cell counts were performed on cytospin-prepared slides (Cytopro Cytocentrifuge; Wescor, Logan, UT) stained with DiffQuick (Siemens, Neward, DE).

### Immunostaining and microscopy of mouse lungs

After BALF collection, whole lungs were harvested and inflated with 1 ml 10% formalin and hung under pressure of 20 cm H_2_O for 24 h for optimal preservation of lung parenchymal architecture. Lung tissue was formalin-fixed, embedded in paraffin, and cut into 5 μm sections. Lung sections were then stained for detection of neutrophils and B-cells using anti-Ly-6B.2 and anti-CD45.R antibodies respectively, according to antibody-specific staining techniques as previously described [24]. Lung lesions (histopathology) in tissue sections were semi-quantitatively scored for severity of alveolitis (infiltration of neutrophils in alveolar parenchyma) and perivascular/peribronchiolar lymphoid cell infiltrates (ectopic lymphoid aggregates) by a veterinary pathologist blinded to treatment conditions (JH), following defined histopathological criteria previously described in detail [24]. Mucous cell in bronchiolar airway epithelium were microscopically detected by periodic acid Schiff (PAS) histochemical staining for intracellular mucosubstances. Severity of mucous cell metaplasia in bronchiolar epithelium was likewise semi-quantitatively scored in PAS-stained lung sections from each animal [24]. In brief, the severity of lung lesions were scored according to the percentage of total lung tissue affected, i.e. severity score 0 for no microscopic findings, 1 for < 10% lung tissue involvement, 2 for 10–25%, 3 for 26–50%, 4 for 51–75%, and 5 for > 75% [24].

To identify mast cells, lung sections were stained for tryptase, and quantification of tryptase^+^ cells per entire lung section was performed using Definiens software with the evaluator blinded to treatment conditions. To assess collagen, Masson’s Trichome staining was performed. Following staining, slides were scanned with iScan Coreo Au (Ventana, Tucson, AZ) slide scanner by the UNMC Tissue Sciences Facility and converted into digital format. Quantification of collagen was performed using ImageJ software after deconvolution and thresholding (Rasband, W.S., Image J, U.S. National Institutes of Health, Bethesda, MD, https://imagej.nih.gov/ij/, 1997–2016) using methods previously described [25, 26].

In the separate studies where animals received repetitive ODEs, rested for 4 weeks and then rechallenged, lung sections were stained for H&E and confocal imaging. For confocal imaging, sections were stained for B cells using ALEXA FLUOR™ 488 conjugated Rat anti-Mouse CD45R (BD Biosciences, San Jose, CA) and macrophages using ALEXA FLUOR™ 594 conjugated rabbit anti-CD68 polyclonal antibody (Bioss Antibodies, Woburn, MA). ALEXA FLUOR™ 488 conjugated Rat IgG and ALEXA FLUOR™ 594 conjugated rabbit IgG (Bioss) were used as isotype controls as previously described [22]. Images were obtained using a Zeiss 510 Meta Confocal Laser Scanning Microscope and analyzed using Image J software (NIH).

### Serum IgE levels

Whole blood was collected from the axillary artery at the time of euthanasia in BD Microtainer tubes (Becton, Dickinson, and Company, Franklin Lakes, NJ), centrifuged for 2 min at 6000 x g and supernatant collected. Serum IgE levels were quantified according to manufacturer’s instructions using ELISA (R&D Systems, Minneapolis, MN).

### Lung homogenate mediator and tight junction analysis

Following BALF collection and vascular perfusion, half of the lung was homogenized in 500 μl of sterile PBS with a gentle MACS dissociator (Miltenyi Biotec, Bergisch Gladbach, Germany) and cell-free supernatant stored at − 80 °C. Levels of the alarmin IL-33, the anti-inflammatory cytokine IL-10, and epidermal growth factor receptor agonist amphiregulin (AREG; involved in lung repair/recovery processes [27]) were quantified by ELISA (Quantikine kit from R&D Systems, Minneapolis, MN) with lower limit of detection of 6.85, 12 and 20 pg/ml. To broadly explore an array of cytokines/chemokines potentially involved in mediating environmental ODE-induced inflammatory lung disease, a custom 18-plex Mouse Magnetic Luminex kit was utilized, which measured CCL2, CCL3, CCL4, CCL5, CCL7, CCL8, CCL11, CCL19, CCL22, CXCL1, CXCL10, IL-4, IL-5, IL-13, periostin, MMP-12, S100A8, and CH13-LI. Protein levels were determined by Bradford assay.

Expression of the tight junction integral proteins claudin-1, − 2, − 3, − 4, − 7, and occludin was investigated by real-time PCR, as previously described, [28] with forward and reverse primer sequences detailed in Additional File 1. Immunoblot analysis was performed using antibodies against claudin-3 (#PA5–16867; Invitrogen), claudin-4 (#PA5–32354, Invitrogen), and occludin (#40–4700; Invitrogen) based on their differential expression in qRT-PCR. Immunoblot analysis of pSTAT3 (#9145S; Cell signaling technology) was included as a control as previously described [29]. Signals were detected using an enhanced chemiluminescence detection kit (Amersham Biosciences). Equal protein loading was determined by re-probing with an anti-β actin antibody (#A2228-100UL; Sigma) after stripping each membrane.

### Statistical methods

Data are presented as means and SEM. A sample-size calculation was estimated from a previous single ODE exposure in WT and MyD88 KO murine study [10], whereby we calculated a sample size of *N* = 3 in each group, to achieve 80% power at the 0.05 level of significance to detect a difference in airway neutrophil influx, assuming a mean (SD) of 11.15 × 10^5^ (4.5 × 10^5^) for WT + ODE and a mean of 0.02 × 10^5^ (0.0065 × 10^5^) for KO + ODE. The experimental groups were run with sex- and age-matched litter mates as available, generally over-sampled, and subsequently pooled. Sample size for each outcome listed in figure legends reflects sample quantity and quality available from the pooled studies. To detect significant differences among groups, a one-way ANOVA was performed, and in the event *p* < 0.05 by ANOVA across groups, a non-parametric Mann-Whitney test was used to determine statistical difference between two groups. Prism software (version 7.0c; GraphPad Software, La Jolla, CA) was used. Significance was accepted at *p*-values < 0.05.

## Results

### MyD88-deficient mice demonstrate a significantly reduced airway inflammatory response following repetitive ODE exposure

**Fig. 2:**
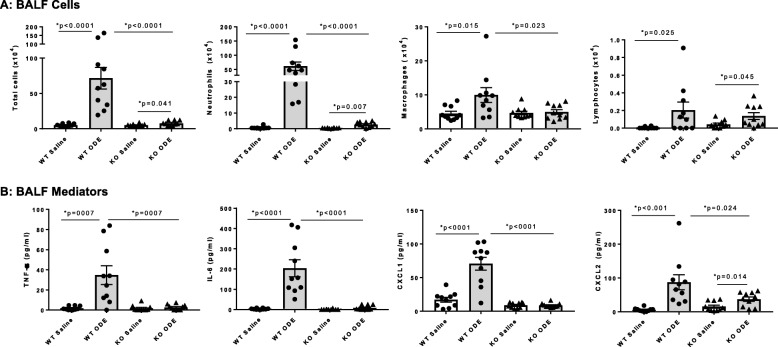
Decreased airway inflammatory response in MyD88 KO mice following repetitive exposure to ODE for 3 weeks. Wild-type (WT) and MyD88 knock-out (KO) mice were treated i.n. with saline or ODE daily for 3 weeks, whereupon bronchoalveolar lavage fluid (BALF) was collected five hours following final exposure. **Panel a**, Total cells, neutrophils, macrophages, and lymphocytes were increased in ODE-treated WT mice, and this response was significantly reduced in MyD88 KO animals. **Panel b**, ODE-induced TNF-α, IL-6, and murine neutrophil chemoattractants (CXCL1 and CXCL2) were reduced or abrogated in MyD88 KO mice. Scatter plots show mean with SEM; *N* = 10 mice/group (*N* = 6 male, *N* = 4 female) from 3 independent experiments

The airway inflammatory response to repetitive daily exposure to ODE for 3 weeks was strikingly reduced in MyD88 KO mice as compared to WT animals. BALF total cells, neutrophils, macrophages, and lymphocytes were increased in ODE-challenged WT mice, and there were significant reductions in total cells, neutrophils, but not lymphocytes, in MyD88 KO animals (Fig. 2a). There was no significant influx of eosinophils detected among the groups (data not shown). Additionally, ODE-induced TNF-α, IL-6, and murine neutrophil chemoattractants (CXCL1 and CXCL2) were either reduced or completely abrogated in MyD88 KO mice (Fig. 2b).

### MyD88-deficient mice demonstrate reduced ODE-induced neutrophilic alveolitis without a reduction in perivascular lymphoid infiltrates

**Fig. 3:**
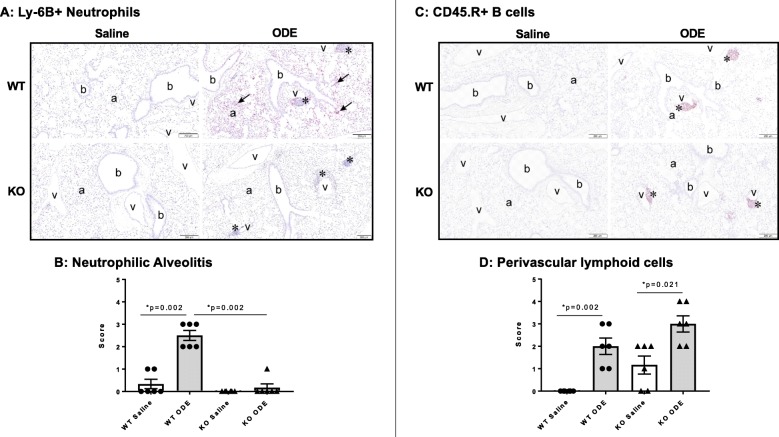
Repetitive ODE exposure-induced neutrophilic alveolitis, but not perivascular lymphoid cell aggregates, are reduced in MyD88 KO mice. Wild-type (WT) and MyD88 knock-out (KO) mice were treated i.n. with saline or ODE daily for 3 weeks, whereupon lung tissues were collected, formalin-fixed, and paraffin embedded. Lung sections (5 μm) were stained for neutrophils (**a**, Ly-6B.2^+^) and B cells (**c**, CD45.R^+^) with representative images for each treatment group shown. Key: b, bronchiolar airway; a, alveolar parenchyma; v = blood vessel; asterisk, lymphoid aggregates, and arrow Ly-6B.2+ neutrophils (scale bar is 200 μm). Lung sections were semi-quantitatively scored from 0 to 5 (see *Methods* section) for neutrophilic alveolitis (**b**) and perivascular lymphoid cells (**d**). Scatter plots (**b, d**) depict mean with SEM; N = 6 mice/group (*N* = 4 male, *N* = 2 female)

Lung tissue sections from exposure groups were stained for neutrophil (Ly-6B.2) and B-cell (CD45.R) infiltrates and semi-quantitatively scored as described in the *Methods* section. Repetitive ODE exposure was found to induce neutrophilic alveolitis and perivascular B cell infiltrates in WT mice (Fig. 3a-d). This alveolitis response to repetitive ODE exposure was reduced in MyD88 KO mice, but the perivascular lymphoid cell response (ectopic lymphoid aggregates) was not affected (Fig. 3a-d).

### MyD88-deficient mice have increased ODE-induced mucus cell metaplasia

**Fig. 4:**
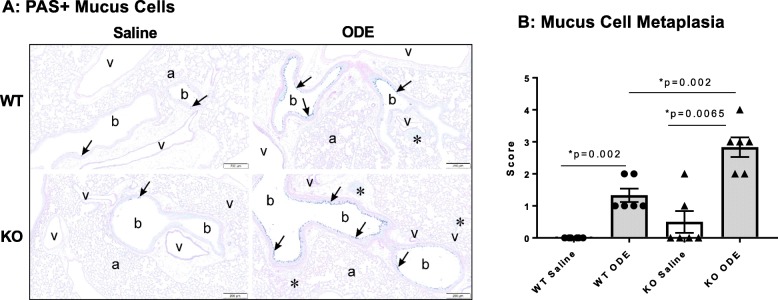
Mucous cell metaplasia is increased in MyD88 KO mice repetitively exposed to inhalant ODE. Wild-type (WT) and MyD88 knock-out (KO) mice were treated i.n. with saline or ODE daily for 3 weeks, whereupon lung tissues were collected, formalin-fixed, and paraffin embedded. Lung sections (5 μm) were PAS-stained for mucus cell determination with representative images for each treatment group shown (**a**). Key: b, bronchiolar airway; a, alveolar parenchyma; v = blood vessel; asterisk, lymphoid aggregates, and arrow denotes positive PAS stained mucus cells (scale bar is 200 μm). Lung sections were semi-quantitatively scored from 0 to 5 for mucus cell metaplasia with scatter plot (**b**) depicting means with SEM; *N* = 6 mice/group (*N* = 4 male, N = 2 female)

It has been previously shown that repetitive ODE exposure for 1 week induces mucus cell metaplasia that is further augmented in MyD88 KO animals [14]. In the present study, mucous cell metaplasia as evident by PAS^+^ staining was increased after repetitive ODE exposure for 3 weeks in both MyD88 WT and KO mice as compared to saline control, and this response was significantly augmented in the ODE-treated MyD88 KO as compared to the ODE-treated WT mice (Fig. 4a-b).

### Lung mast cells, lung IL-33 and serum IgE levels are increased following repetitive ODE exposure and differentially modulated by MyD88

**Fig. 5:**
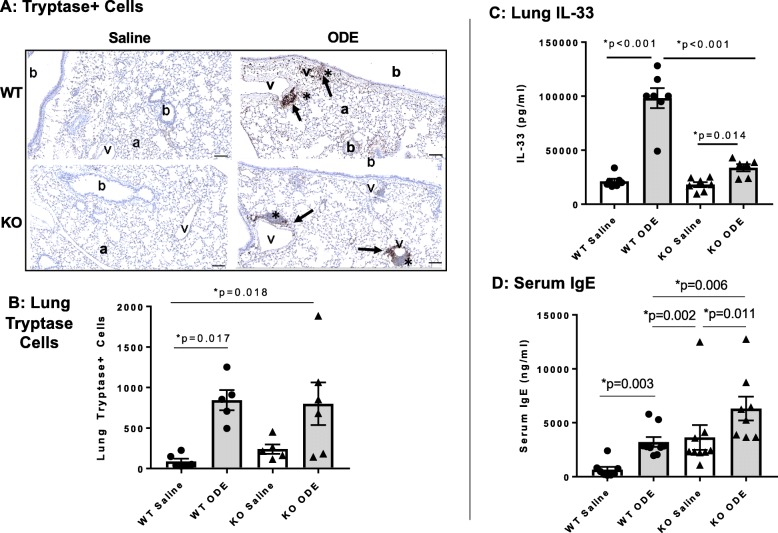
Tryptase^+^ lung mast cells, serum IgE and lung IL-33 levels are increased following repetitive ODE exposure and variably modulated by MyD88. WT and MyD88 KO mice were treated i.n. daily for 3 weeks with saline or ODE whereupon lung sections (5 μm thick) were stained for tryptase with representative images for each treatment group shown **a**. Key: b, bronchiolar airway; a, alveolar parenchyma; v = blood vessel; asterisk, cellular aggregates; and arrow denotes positive tryptase stained cells in cellular aggregates (scale bar is 100 μm). **b**, Scatter plot depicts means with SEM of tryptase positive cells per entire lung section as quantified by Definiens software with *N* = 5–6 mice/group. **c,** Serum IgE levels quantified by ELISA with *N* = 8–9 mice group. **d**, ODE-induced lung homogenate IL-33 levels were reduced in MyD88 KO mice with *N* = 6–7 mice/group

Repetitive ODE exposure increased tryptase^+^ lung mast cells in both WT and MyD88 KO mice, predominately observed within the perivascular aggregates (Fig. 5a-b). There was no difference between ODE-treated WT and KO animals. We then sought to quantitate levels of IL-33, an alarmin cytokine involved in mast cell activation and mucin expression [30]; and moreover, IL-33R signals via MyD88 to potentially reflect an autocrine/paracrine action [31]. ODE exposure significantly induced lung IL-33 levels in WT and KO animals as compared to saline, but this response was significantly reduced in ODE-treated MyD88 KO mice as compared to WT mice (Fig. 5c). It has been previously demonstrated that ODE exposure slightly, but significantly increases serum IgE levels [32]. In these studies, serum IgE levels were increased in both ODE-treated WT and MyD88 KO mice in comparison to their respective saline-treated controls (Fig. 5d). Serum IgE levels were significantly increased in saline control MyD88 KO mice as compared to saline control WT mice, which is consistent with a prior report showing elevated levels of serum IgE in saline control treated MyD88 KO animals [33]. However, a separate study showed decreased IgE levels in MyD88 KO animals [34, 35].

### Repetitive ODE exposure increases mediators involved in neutrophil, lymphocyte, monocyte, and mast cell recruitment as well as factors involved in tissue remodeling, which are largely attenuated in MyD88 deficient mice

To further delineate the immunophenotype induced by ODE, we investigated a number of inflammatory, allergy, and non-allergy mediators by cytokine array analysis **(**Table 1; significant differences across treatment groups are in bold). Repetitive exposure to ODE did not induce an increase in the Th2 cytokines IL-4, IL-5, and IL-13 which are known to be involved in the classical allergy response and agrees with the lack of MyD88 involvement in regulating serum IgE levels. However, in both WT and MyD88 KO mice repetitively exposed to ODE there were significant increases in CCL2, CCL3, CCL4, CCL5, and CCL7. These mediators are known to be involved with recruitment of neutrophils, lymphocytes, monocytes, and mast cells. Moreover, these responses were attenuated in MyD88 KO mice as compared to WT animals, confirming the reduced recruitment of these effector cells in the lungs of MyD88 KO mice following ODE. Additionally, ODE exposure increased levels of neutrophilic inflammatory mediator S100A8 and tissue remodeling factors including periostin, MMP-12, CHI3-L1, and amphiregulin (AREG) in WT mice. These mediators were not induced in ODE-challenged MyD88 KO mice. There was no significant difference in total protein levels in lung homogenates across treatment groups.

**Table 1:** Levels of mediators in lung tissue homogenates screened by treatment group

Mediator	Action	WT Saline	WT ODE	KO Saline	KO ODE
**CCL2**	Attracts monocytes, memory T cells, DCs	181.5 ± 27.7	625.2 ± 18.4 **	371.1 ± 87.3	557.0 ± 96.0*
**CCL3**	Attracts neutrophils	21.5 ± 4.5	874.5 ± 198.3***,###	43.3 ± 11.9	100.4 ± 36.4*
**CCL4**	Attracts NK cells, monocytes	37.5 ± 5.3	140.6 ± 23.8***, ###	37.5 ± 3.7	**48.8 ± 7.5**
**CCL5**	Attracts T cells, eosinophils, basophils	252.1 ± 67.5	1410 ± 450.5**	545.1 ± 98.9	2519 ± 600.2*
**CCL7**	Attracts monocytes, activates MΦ	34.4 ± 9.2	552.5 ± 117.3***,##	27.5 ± 5.7	150.5 ± 53.7*
CCL8	Attracts mast cells, eosinophils and basophils, monocytes, T cells, NK cells	356.3 ± 163.3	1620 ± 620.6	591.8 ± 112.4	1181 ± 215.2
CCL11	Attracts eosinophils	94.2 ± 39.7	177.4 ± 52.3	77.8 ± 9.3	97.7 ± 10.7
CCL19	Attracts T and B cells	14.6 ± 2.5	18.7 ± 1.1	15.9 ± 1.4	22.2 ± 6.5
**CCL22**	Attracts Th2 and Treg	117.5 ± 38.3	432.1 ± 52.6**	208.5 ± 62.4	335.4 ± 88.3
**CXCL1**	Attracts neutrophils	62.3 ± 20.6	713.1 ± 211.3**,##	30.2 ± 6.2	47.8 ± 11.0
**CXCL10**	Attracts monocytes/macrophages, T cells, NK cells, DCs	44.2 ± 12.1	131.6 ± 29.3*	40.8 ± 3.1	107.2 ± 38.9
IL-4	Th2, allergy	236.2 ± 25.1	173.5 ± 16.3	303.9 ± 24.5	273.8 ± 5.1
IL-5	Eosinophil recruitment	3.4 ± 0.9	3.0 ± 0.2	2.6 ± 0.1	2.8 ± 0.1
IL-13	Regulates IgE, mucus	246.2 ± 14	173.5 ± 16.3	303.9 ± 24.5	273.8 ± 5.1
**Periostin**^**a**^	Tissue remodeling	9.99 ± 1.11	21.14 ± 2.71***, #	8.91 ± 9.88	12.42 ± 1.40
**MMP-12**	Tissue remodeling	757.7 ± 337.9	13,351 ± 5086**,##	647.8 ± 133.5	854.5 ± 196.1
**S100A8**^**a**^	Neutrophilic inflammation	229.9 ± 61.1	652.7 ± 66.4***,###	172.5 ± 33.8	215.4 ± 9.6
**CHI3-L1**^**a**^	Inflammation and tissue remodeling	291.5 ± 42.1	620.0 ± 9.5*, #	339.4 ± 67.6	468.9 ± 70.0
**AREG**	Tissue remodeling and repair	102.3 ± 11.6	221.8 ± 23.5**	122.4 ± 3.7	161.5 ± 19.0
Total Protein^a^		7.50 ± 0.47	8.81 ± 0.41	6.47 ± 0.83	5.61 ± 1.46

Mean ± SEM values shown in pg/ml; except ^a^values are ng/ml. N = 4 mice/ group (2 males/2 females). Statistical difference denoted (*p < 0.05 **p < 0.01, ***p < 0.001) vs. saline. Statistical difference between WT ODE and KO ODE denoted (#*p* < 0.05, ##*p* < 0.01, ###*p* < 0.001). Differences are in bold

Protein levels in the BALF increased in ODE treated WT mice, but not MyD88 KO animals, suggesting modest disruption in alveolar permeability [36]. Specifically, mean ± SEM of BALF protein levels were significantly different (*p* = 0.019) between WT saline: 140.1 ± 13.4 μg/ml vs. WT ODE: 204.8 ± 19.9 μg/ml, but were not significantly different (*p* > 0.05) between MyD88 KO saline: 142.8 ± 7.2 μg/ml vs. KO ODE: 158.3 ± 10.6 μg/ml.

### Repetitive ODE exposure augments the expression of several tight junction proteins in the lung that are differentially regulated by MyD88

**Fig. 6:**
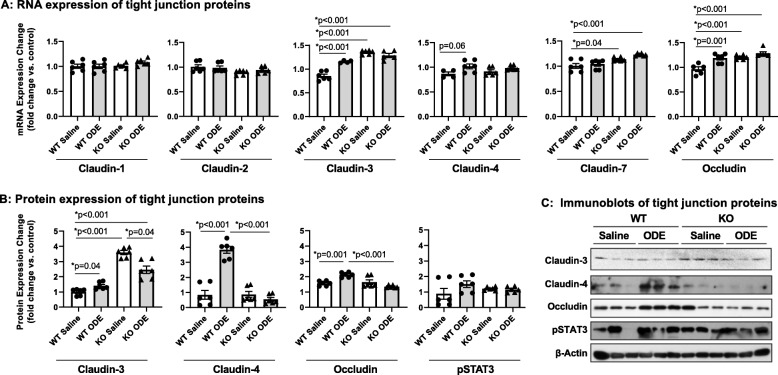
Repetitive ODE exposure increases expression of several tight junction proteins known to be upregulated in inflamed/injured lung in WT mice but not in MyD88 KO mice. WT and MyD88 KO mice were treated i.n. daily for 3 weeks with saline or ODE. **Panel a,** Expression of tight junction mRNA was measured by real-time quantitative PCR and are reported as fold-changes normalized to control. **Panel b**, Quantification of tight junction protein expression in ODE-treated mice compared to control mice as determined by the immunoblot presented in **Panel c**. Scatter plots demonstrate mean with standard error bars of *N* = 3 animals per group with 2 replicates per sample

Studies have reported a potential adaptive role for specific tight junction proteins in the inflamed and injured lung, in particular claudin-4 [37, 38]. Our initial RT-qPCR analysis supported trends (*p* = 0.06) for increased gene expression of claudin-4 in ODE-challenged WT mice as well as claudin-3 and occludin (Fig. 6a). In the same samples, expression of claudin-1, − 2 and − 7 were unaltered supporting the specificity of the above outcomes. Immunoblot analysis further demonstrated robust and significant (*p* < 0.001) upregulation of claudin-4 in ODE-challenged WT mice as compared to WT saline (Fig. 6b-c). There were also significant increases in claudin-3 and occludin with ODE exposure as compared to saline in WT animals (Fig. 6b-c). In comparison, gene and protein expression of these tight junction proteins were not increased in ODE-treated MyD88 KO animals as compared to saline-treated KO mice. As compared to WT saline, gene expression of claudin-3, claudin-7, and occludin were increased in MyD88 KO animals (with/without ODE), and by protein measurements, only claudin-3 was increased in these KO animals. The significance of this basal difference in MyD88 KO animals is unknown.

### MyD88 is required for eliciting immune responses upon rechallenge after a period of rest following repetitive ODE exposure

**Fig. 7:**
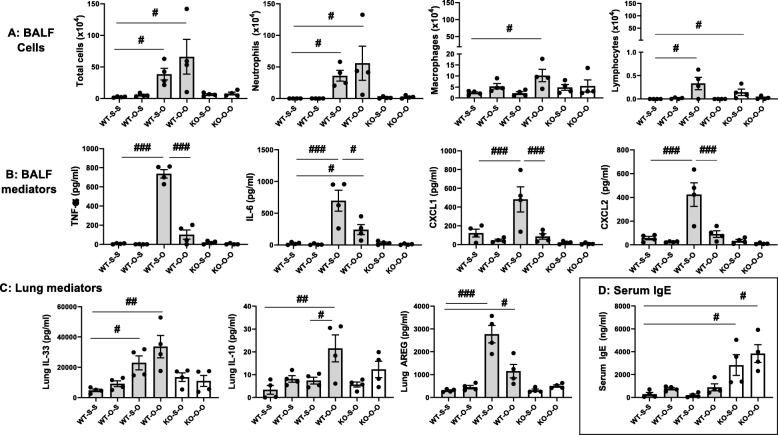
An airway inflammatory adaptive response following ODE rechallenge in WT mice after a 4 week recovery from repetitive exposures with minimal responsiveness observed in MyD88 KO mice. WT and MyD88 KO mice were repetitively exposed to saline or ODE for 3 weeks followed by a 4 week rest/recovery phase and then subsequently rechallenged once with saline or ODE. Scatter dot plots demonstrate mean with standard error bars with treatment groups denoted as S-S (saline repeated exposure-saline rechallenge), O-S (ODE repeated exposure-saline rechallenge), S-O (saline repeated exposure-ODE rechallenge), O-O (ODE repeated exposure-ODE rechallenge). **Panel a:** Total cells, neutrophils, macrophages, and lymphocytes in BALF. **Panel b**: Proinflammatory cytokines/chemokines quantified in cell-free BALF. **Panel c**: Mediator levels quantified in cell-free lung homogenates. **Panel d**: Serum IgE levels. Statistical significance (#*p* < 0.05; ##*p* < 0.01; ###*p* < 0.001) vs. saline or denoted by line. *N* = 4 mice group (2 female/2 male)

Total cells and neutrophils were increased in the BALF of WT mice pretreated with saline or ODE and then challenged once with ODE (WT-S-O and WT-O-O) as compared to saline control (WT-S-S) (Fig. 7a). There was no difference between WT mice rechallenged with ODE after repetitive saline (WT-S-O) and WT mice rechallenged with ODE after repetitive ODE (WT-O-O) (Fig. 7a). BALF macrophages were significantly increased only in WT mice pre-treated with ODE and then rechallenged with ODE (WT-O-O). BALF lymphocytes were significantly increased in WT mice pre-treated with saline and then rechallenge with ODE (WT-S-O). There were no significant increases in total cells, neutrophils, or macrophages in MyD88 KO mice pretreated with either saline or ODE and then rechallenged with ODE (KO-S-O or KO-O-O). However, lymphocytes were increased in MyD88 KO mice pretreated with saline and then rechallenged with ODE (KO-S-O) as compared to KO-O-O mice (mice pretreated with ODE and then rechallenged with ODE).

A different pattern of responses was observed with ODE-induced inflammatory mediators (i.e. TNF-α, IL-6, CXCL1 and CXCL2) (Fig. 7b). Namely, these inflammatory mediators were robustly increased in WT mice pretreated with saline and rechallenged with ODE (WT-S-O). However, these responses were strikingly attenuated in mice pretreated with ODE and rechallenged with ODE (WT-O-O). The MyD88 KO mice, regardless of pretreatment with saline or ODE, failed to mount an inflammatory mediator response to the ODE rechallenge.

### Lung mediators and serum IgE were variably regulated in mice rechallenged with ODE following the prolonged 4-week recovery phase

Lung homogenates from the same treated WT and MyD88 KO mice were analyzed for several mediators. Lung IL-33 levels were increased in WT mice pretreated with saline or ODE and then challenged once with ODE (WT-S-O and WT-O-O) as compared to saline controls (Fig. 7c), and there was no significant difference between WT-S-O and WT-O-O. There was no detectable IL-33 response in MyD88 KO mice. Levels of the anti-inflammatory cytokine IL-10 in the lung were increased only in the WT mice pretreated with ODE and then rechallenged once with ODE (WT-O-O) (Fig. 7c). The repair/recovery mediator AREG was increased in WT mice pretreated with saline or ODE and rechallenged with ODE (WT-S-O or WT-O-O) as compared to mice rechallenged with saline (WT-S-S). There was a non-significant (*p* = 0.057) decrease in AREG release in WT mice pretreated with ODE and rechallenged with ODE (WT-O-O) as compared to WT saline pretreated and ODE rechallenged (WT-S-O) (Fig. 7c). Lung IL-10 and AREG levels were not significantly modulated in MyD88 KO mice. Lastly, we quantitated serum IgE levels in these same mice, and serum IgE levels were increased only in the MyD88 KO mice (regardless of pretreatment conditions) and were unchanged across the WT mice treatment groups (Fig. 7d).

### Exacerbated lung histopathology occurs with ODE rechallenge following a 3-week exposure and 4-week rest/recovery phase

**Fig. 8:**
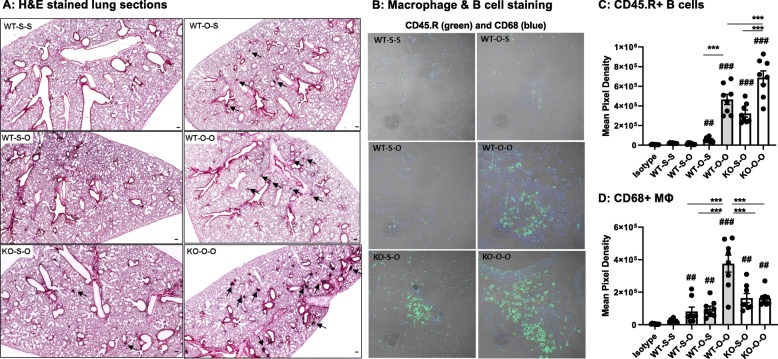
Lung histopathology accentuated with rechallenge following a 4 week recovery after repetitive ODE exposure. WT and MyD88 KO mice were repetitively exposed to saline or ODE for 3 weeks followed by a 4 week rest/recovery phase and then subsequently re-challenge once with saline or ODE. **Panel a**, Lung sections (5 μm) were H&E stained with representative images shown from each group of 4 animals/group (scale denotes 100 μm; 2x magnification). Arrows denote lymphocytic aggregates. **Panel b**, Confocal images of lung tissues stained for B cells (CD45.R; green) and macrophages (CD68; blue) shown at 40x magnification with selected images focused on aggregates where available. Scatter dot plots demonstrate mean with standard error bars of mean pixel density representing two regions of interest (ROIs) from each mouse (N = 8 ROI/group) depicting CD45.R^+^ B cells **c** and CD68^+^ Macrophages **d**. Treatment groups denoted as S-S (saline repeated exposure-saline rechallenge), O-S (ODE repeated exposure-saline rechallenge), S-O (saline repeated exposure-ODE rechallenge), and O-O (ODE repeated exposure-ODE rechallenge). Statistical significance denoted as ##*p* < 0.01, ###*p* < 0.001 vs. WT-S-S; and **p < 0.01, ***p < 0.001 denoted between groups (line)

Microscopically, there was a persistence of perivascular lymphoid infiltrates after repetitive ODE in both WT and MyD88 KO animals following 4-week rest (Fig. 8a**;** isotype control antibody staining shown in Additional File 2). These perivascular ectopic lymphoid aggregates were more pronounced in the MyD88 KO animals repetitively treated with ODE and rechallenged with ODE (KO-O-O) as compared to the WT-O-O treated mice. CD45.R^+^ B cells were prominent in ODE-induced perivascular lymphocytic aggregates (Fig. 8b). There was an increase in B cell infiltrates in WT and KO mice pretreated with ODE and rechallenged with ODE (O-O) as compared to animals rechallenged with saline (O-S). B cell infiltrates in aggregates were increased in KO mice as compared to WT (Fig. 8c). CD68^+^ macrophages were increased with ODE treatment (Fig. 8b, d) and macrophage recruitment was the highest in the WT-O-O treatment group.

## Discussion

Organic dusts from agricultural environments contain an array of bacterial motifs recognized to play important roles in mediating airway inflammatory disease [1], and we have previously shown that MyD88 has a fundamental role in the acute inflammatory response to organic dust [10]. Here we demonstrate that MyD88 remains critical to mediating the airway inflammatory response following daily, repetitive exposures for 3 weeks as demonstrated by neutrophil influx and increases in a wide array of inflammatory mediators that were nearly abrogated in MyD88 KO mice. MyD88 was not responsible for lymphocyte recruitment as lymphocytes in the BALF and B cell infiltrates in the lung parenchyma were not reduced in ODE-challenged MyD88 KO mice. Mucus cell metaplasia was augmented in MyD88 KO animals, which cannot be explained by alterations in inflammatory mediators classically implicated in mucus cell biology, such as IL-6, IL-4, IL-13, and IL-33. Repetitive ODE exposure also induces a prolonged adaptation response. Despite a 4-week rest/recovery phase following repetitive ODE exposures, there was significant reduction in inflammatory mediator levels, but not neutrophil influx, in WT animals following a single rechallenge. These findings were also dependent upon MyD88. Upregulated expression of claudin-4, − 3 and occludin proteins in ODE-challenged mice further support this concept as these proteins are known to be upregulated in response to lung inflammation/injury and render adaptive roles [37, 38]. Together, these findings illustrate the complex role of the innate immune MyD88 signaling pathway with repetitive organic dust exposures and rechallenge.

Agriculture organic dust exposures are well-established to induce a neutrophilic as opposed to an eosinophilic influx with a corresponding release of pro-inflammatory mediators including TNF-α, IL-6, and neutrophil chemoattractants CXCL1 and CXCL2 [1]. Expanding on these findings, repetitive ODE exposures were also found to induce IL-33, the neutrophil mediator S100A8, and several factors involved with neutrophil, lymphocyte, monocyte, and mast cell recruitment (i.e. CCL2, CCL3, CCL4, CCL5, and CCL7). The classic Th2 cytokines implicated in allergic responses (i.e. IL-4, IL-5, and IL-13) were not induced. Further, tissue remodeling/pre-fibrosis/repair mediators including periostin, MMP-12, and CHI3-L1 as well as amphiregulin (AREG) were increased after repetitive ODE. Nearly all these responses were MyD88-dependent. Thus, not only is the innate MyD88 signaling pathway central to acute ODE induced inflammatory responses, these new studies suggest that there is not a compensatory response with repeated daily exposures over time regarding these multiple inflammatory attributes. Furthermore, MyD88 was central to mediating the airway inflammatory response following a prolonged rest period and subsequent rechallenge. Therefore, targeting MyD88 could represent a strategy in occupationally exposed workers to reduce airway inflammatory disease burden.

Various agriculture organic dust exposures in humans and mice also induce lymphocytic responses typified by a Th1 and/or Th17 microenvironment in the lung [22, 39, 40]. In the current study, we focused on B cells due to our recent report demonstrating their importance in experimental ODE-induced lung disease [22]. Current studies suggest that MyD88 may be important in resolution processes of environmentally triggered lung disease. Whereas there was no difference in B cell infiltrates with repetitive ODE exposure between WT and MyD88 KO animals, B cell infiltrates were increased in mice lacking MyD88 at 4 weeks post-recovery and a one-time rechallenge further augmented B cell infiltrates in the MyD88 KO mice. Others have also found that absence of MyD88 results in unresolved pulmonary infiltrates following long-term radiation-induced lung injury, [41] silica-induced fibrosis [42], and bacterial pneumonia [43]. The explanation for the persistence of lung infiltrates has been attributed to the generation of regulatory cells triggered through TLR/MyD88 signaling [41–43].

It is likely that macrophages and IL-10 contribute to the resolution process and response to ODE rechallenge. It is known that depletion of lung macrophages impairs lung recovery following repetitive ODE exposures [44]. In addition, mice lacking scavenger receptor A (CD204), a receptor responsible for enhancing IL-10 production under inflammatory states [45, 46], have impaired recovery [47]. Here, we report that macrophages and IL-10 levels were significantly increased in WT mice pretreated with ODE and rechallenged with ODE, which we interpret to explain the profoundly affected (i.e. reduced) airway inflammatory cytokine response to rechallenge. This reduced response is in contrast to a hypothesis that the lung could have a heightened inflammatory response upon rechallenge as observed in Byssinosis (i.e. endotoxin-mediated disease), whereby textile workers experience worsening dyspnea on Mondays or after vacations [48]. Agriculture organic dusts are complex and not just comprised of endotoxin, and it is likely that the wide diversity of bacterial motif exposures explain the prolonged adaptive/modulated response of the lung microenvironment. Interestingly, others have recognized the benefit of this type of immunostimulatory driver, as inhaled treatment of a synergistic combination of TLR agonists protect mice against a wide array of lethal pneumonias and are being explored for potential therapeutic applications [49–51].

Mucus production and barrier control are additional innate responses to ODE that were found to be governed by MyD88. In this regard, our studies found that ODE exposure specifically increased expression of the tight junctions claudin-4, − 3 and occludin, which were lost in MyD88-deficient mice. Increased claudin-4 has been reported in the inflamed and injured lung, and has been associated with adaptive (regulatory) responses, as inhibiting claudin-4 function in mice promoted the severity of lung injury [37, 38]. We attribute a similar adaptive role for occludin. These findings emphasize the potential communication between MyD88 signaling and barrier function in regulating the lung airway homeostasis and injury responses as these responses were absent in ODE-challenged MyD88 KO mice. Curiously, claudin-3 expression was dramatically increased in MyD88 KO mice. While we currently do not understand the implications of this finding, studies have reported independent roles for claudin-3 and claudin-4 in regulating lung epithelial barrier properties despite their high sequence similarity [52]. The functional consequence of these changes in tight junction protein expression remains unclear. There was evidence to support that alveolar permeability was impacted with ODE exposure as protein levels were modestly increased in the lavage fluid of ODE-treated WT, which was inhibited in MyD88 KO mice. Collectively, these studies would support new directions to investigate the functional role of tight junctions through potentially utilizing genetically modified mice or through pharmacologic directed approaches.

In addition, AREG can promote the lung repair process, but prolonged overexpression can also lead to fibrotic remodeling [27, 53, 54]. It was recently reported that ODE increases AREG levels, and importantly, a 3-day treatment period with lung-delivered recombinant AREG hastened lung resolution/recovery following dust exposure [55]. Here, we confirm increased AREG levels with repetitive ODE exposure and demonstrate that upon re-exposure to ODE, AREG levels are dampened. These responses were inhibited in MyD88 KO animals. It is possible that the absence of an AREG response could be potentially important in explaining the persistence of the lymphoid pathology demonstrated in the MyD88 KO animals. It is also recognized that intranasal inhalation delivery method has consistently resulted in a predominance of central airway involvement as evident by pathology being focused around the bronchopulmonary compartment. However, with repeated exposures, the lung parenchyma or interstitial space is increasingly involved, but to a lesser degree as marked by increase in neutrophilic alveolitis. This neutrophilic alveolitis was dependent upon MyD88.

The exaggerated mucous cell metaplasia in mice lacking MyD88 following repetitive ODE for 3 weeks is consistent with prior work demonstrating enhanced mucus cell metaplasia after 1 week of exposure [14]. In this previous work, there were no differences in mucous cell numbers, but negative regulation of Muc5ac transcription by MyD88 in secretory cells was demonstrated [14]. In the current report, we demonstrated that this response remains uncoupled from classic inflammatory mucus cell metaplasia mediators, including neutrophils, IL-33, Th2 cytokines, and various pro-inflammatory cytokines. It is recognized that full-length IL-33 has modest biological activity and that its activity can be rapidly enhanced by removal of N-terminus by serine proteases released from neutrophils and mast cells as well as rapidly inactivated by disulfide bonding of critical cysteine residues. We reported on full-length IL-33, and it may be warranted to investigate processed forms of IL-33 [56]. Increased numbers of airway mast cells are also associated with enhanced mucin [57], and although ODE increased mast cells, there was no difference in MyD88 KO and WT animals. Interestingly, IgE levels were inherently increased in MyD88 KO mice and further augmented with ODE. Therefore, it is possible to speculate that an IgE-dependent mechanism, presumably through local mast cell activation, could be playing a potential role in ODE-induced mucus cell metaplasia. However, using tryptase as a marker of mast cell activation, we were unable to detect any differences across treatment groups in serum or BALF tryptase levels (data not shown). It might be warranted in future studies to directly target IgE in regulating mucin in this non-eosinophilic, neutrophilic-associated airway disease, as targeting IgE is a clinically available therapeutic approach.

## Conclusions

Collectively, our findings demonstrate that MyD88 remains essential to the airway inflammatory response following repetitive and rechallenges with ODE exposures. The MyD88 pathway strongly regulates neutrophilic and pro-inflammatory responses to ODE that predominately impacting the central airways, but also the alveolar compartment. However, despite these profound reductions with inflammatory mediators, lymphocytic infiltrates and mucin production remains and even to some degree, potentiated, in this MyD88 deficient state. There is a long-lasting adaptation response following ODE exposures with anti-inflammatory features demonstrated upon rechallenge. Exploiting the MyD88 signaling pathway in the lung could represent future preventative and/or therapeutic targets.

## Supplementary information

**Additional file 1.** Real-time PCR forward and reverse sequence primers tight junction proteins expression of mice.

**Additional file 2.** Confocal images of isotype antibody staining of lung sections from WT and MyD88 KO mice repetitively exposed to ODE for 3 weeks, rested for 4 weeks, and then rechallenged once with ODE or saline. Treatment groups denoted as S-S (saline repeated exposure-saline rechallenge), O-S (ODE repeated exposure-saline rechallenge), SO (saline repeated exposure-ODE rechallenge), O-O (ODE repeated exposure-ODE rechallenge).

## Data Availability

The datasets used and/or analyzed during the current study are available from the corresponding author on reasonable request.

## Abbreviations

TLR: Toll-like receptor
COPD: Chronic obstructive pulmonary disease
SPF: Specific-pathogen-free
MyD88: Myeloid differentiation factor 88
ODE: Organic dust extract
KO: Knock-out
WT: Wild-type
PBS: Phosphate buffered saline
BALF: Bronchoalveolar lavage fluid
AREG: Amphiregulin
