# Supplementary material for: MyD88 regulates a prolonged adaptation response to environmental dust exposure-induced lung disease
Source: Respir Res. 2020 Apr 22;21:97. doi: 10.1186/s12931-020-01362-8 (PMC7178993; doi:10.1186/s12931-020-01362-8)
Supplement: Supplementary file 1 — Additional file 1. Real-time PCR forward and reverse sequence primers tight junction proteins expression of mice. [file 12931_2020_1362_MOESM1_ESM.docx]

**Additional File 1.** Real-time PCR forward and reverse sequence primers tight junction proteins expression of mice.

| **Primer** | **Sequence** |
| --- | --- |
| Claudin-1 Forward Primer | TCT ACG AGG GAC TGT GGA TG |
| Claudin-1 Reverse Primer | TCA GAT TCA GCA AGG AGT CG |
| Claudin-2 Forward Primer | GGC TGT TAG GCA CAT CCA |
| Claudin-2 Reverse Primer | TGG CAC CAA CAT AGG AAC TC |
| Claudin-3 Forward Primer | GAT GGG AGC TGG GTT GTAC |
| Claudin-3 Reverse Primer | CGC AGA ATA GAG GAT CTT GGT G |
| Claudin-4 Forward Primer | GTT CAT CGT GGC AAG CAT G |
| Claudin-4 Reverse Primer | CCA TAG GGT TGT AGA AGT CGC |
| Claudin-7 Forward Primer | GAC GTT AGG TTA TTT TCG GTC |
| Claudin-7 Reverse Primer | AAA CGC GTT TCT AAA CGC CG |
| Occludin Forward Primer | GAT TCC TCT GAC CTT GAG TGT G |
| Occludin Reverse Primer | GTT TCA TAG TGG TCA GGG TCC |
