# Supplementary material for: MyD88 regulates a prolonged adaptation response to environmental dust exposure-induced lung disease
Source: Respir Res. 2020 Apr 22;21:97. doi: 10.1186/s12931-020-01362-8 (PMC7178993; doi:10.1186/s12931-020-01362-8)
Supplement: Supplementary file 2 — Additional file 2. Confocal images of isotype antibody staining of lung sections from WT and MyD88 KO mice repetitively exposed to ODE for 3 weeks, rested for 4 weeks, and then rechallenged once with ODE or saline. Treatment groups denoted as S-S (saline repeated exposure-saline rechallenge), O-S (ODE repeated exposure-saline rechallenge), SO (saline repeated exposure-ODE rechallenge), O-O (ODE repeated exposure-ODE rechallenge). [file 12931_2020_1362_MOESM2_ESM.pdf]

**Additional File 2: Isotype antibody staining.**

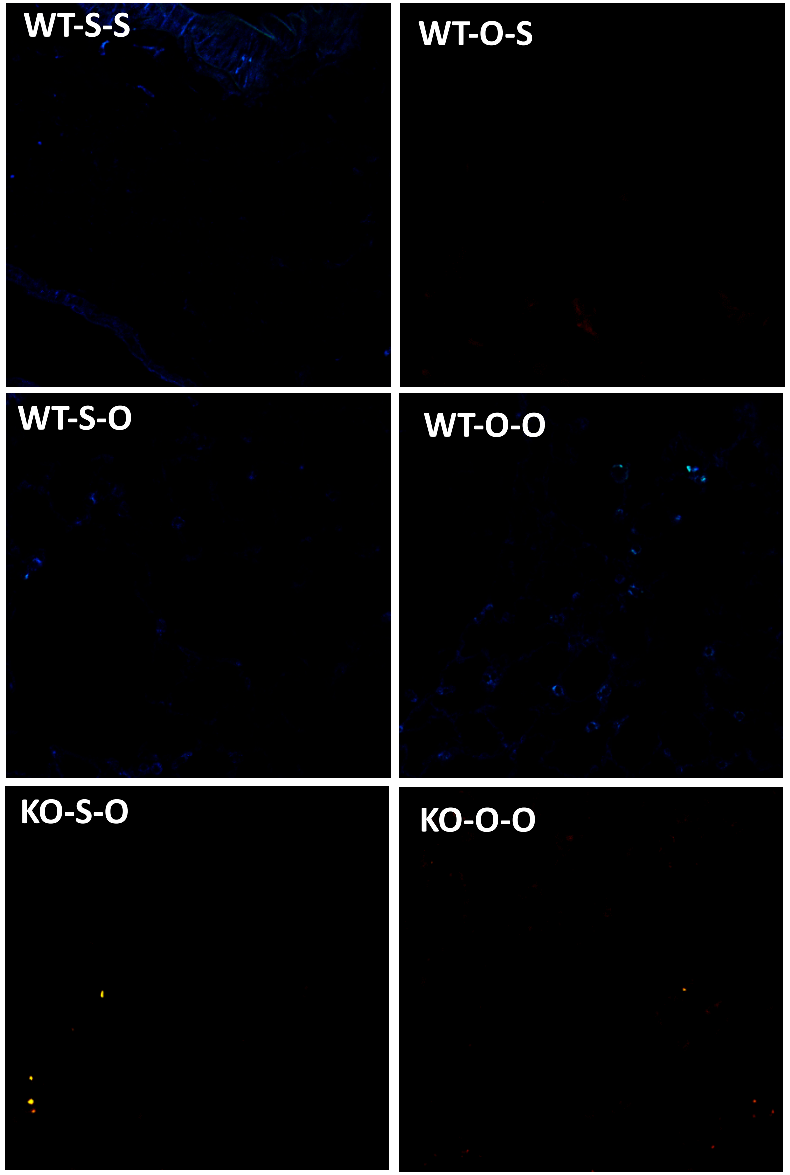


Additional File 2. Confocal images of isotype antibody staining of lung sections from WT and MyD88 KO mice repetitively exposed to ODE for 3 weeks, rested for 4 weeks, and then rechallenged once with ODE or saline. Treatment groups denoted as S-S (saline repeated exposure-saline rechallenge), O-S (ODE repeated exposure-saline rechallenge), S-O (saline repeated exposure-ODE rechallenge), O-O (ODE repeated exposure-ODE rechallenge).
